# Optimizing use of U.S. Ex-PVP inbred lines for enhancing agronomic performance of tropical *Striga* resistant maize inbred lines

**DOI:** 10.1186/s12870-022-03662-1

**Published:** 2022-06-10

**Authors:** Abdoul-Raouf Sayadi Maazou, Melaku Gedil, Victor O. Adetimirin, Wende Mengesha, Silvestro Meseka, Oluyinka Ilesanmi, Paterne A. Agre, Abebe Menkir

**Affiliations:** 1 Pan African University Life and Earth Sciences Institute (Including Health and Agriculture), University of Ibadan, Ibadan, 200284 Nigeria; 2 International Institute of Tropical Agriculture (IITA), PMB 5320, Ibadan, 200001 Nigeria; 3 Department of Crop and Horticultural Sciences, University of Ibadan, Ibadan, 200284 Nigeria

**Keywords:** Exotic germplasm, Ex-PVP inbred lines, Tropical maize breeding, Combining ability, Heterotic groups

## Abstract

**Background:**

Temperate maize inbred lines with expired Plant Variety Protection Act certificates (Ex-PVP) are potential sources of desirable alleles for tropical germplasm improvement. Up to now, the usefulness of the Ex-PVP inbred lines as a potential source of novel beneficial alleles for *Striga hermonthica* resistance breeding to enhance genetic gain in tropical maize has not been reported.

**Results:**

This study was thus conducted to characterize the combining ability of 24 Ex-PVP inbred lines in crosses with two tropical *Striga* resistant inbred testers under *Striga*-infested and non-infested conditions and across three locations for 2 years. Many testcrosses between Ex-PVP inbred lines and the first tester (T1) produced competitive or significantly higher grain yields compared to the hybrid between the two resistant testers under *Striga* infested and non-infested conditions and across multiple test locations. Also, most of the testcrosses with positive heterosis for grain yield and negative heterosis for *Striga* damage and emerged *Striga* count involved T1 as a tester. Our study identified six Ex-PVP inbred lines with positive GCA effects for grain yield under *Striga* infested and non-infested conditions and across multiple test locations. Amongst these, inbred lines HB8229-1 and WIL900-1 also displayed negative GCA effects for emerged *Striga* count and *Striga* damage rating. The inbred line HB8229-1 showed positive SCA effects for grain yield with T2, whereas WIL900-1 had positive SCA effects for grain yield with T1. Over 70% of the Ex-PVP inbred lines were consistently assigned to specific heterotic groups using yield-based classifying methods (mean grain yield and SCA effects).

**Conclusions:**

These results could facilitate systematic introgression of the Ex-PVP inbred lines into the existing *Striga* resistant heterotic groups in IITA. The Ex-PVP inbred lines with positive GCA effects and producing high grain yields in hybrid combinations could be useful parents for enhancing *Striga* resistance and agronomic performance of tropical maize hybrids.

**Supplementary Information:**

The online version contains supplementary material available at 10.1186/s12870-022-03662-1.

## Background

Maize (*Zea mays* L.) is widely cultivated and consumed as a staple food in Africa. The projected increase in population is expected to double the demand for maize in developing countries by 2050 [1]. The increase in demand for maize particularly in sub-Saharan Africa is expected to reach 24% [2]. Although significant progress has been made in breeding maize for higher grain yields, grain yields are still low to meet the increasing demand from consumers. These low grain yields result mainly from parasitic weeds including *Striga*, pest and diseases, and low soil fertility as well as drought and increasing temperature. *Striga hermonthica* (Del.) Benth parasitism poses a major threat to maize production in the savannas of west and central Africa. The parasite can inflict up to 100% grain yield loss, particularly in marginal production areas [3]. The development of maize varieties with polygenic resistance to *S. hermonthica* has been considered central to an integrated management strategy to minimize grain yield losses in farmers’ fields where the parasite is endemic [4–6]. Breeders at the International Insititute of Tropical Agriculture (IITA) have, therefore, been developing maize germplasm with polygenic resistance to *S. hermonthica* to provide durable protection against diverse parasite populations.

In search for sources of polygenic resistance to *S. hermonthica*, IITA maize breeders screened many adapted tropical maize inbred lines, hybrids and landraces as well as wild relatives of maize under natural and artificial *S. hermonthica* infestation in the field and the greenhouse. These led to the development of maize inbred lines and hybrids with a consistent expression of resistance to the parasite [5–10]. One of the inbred lines (TZI25), which is an adapted backcross conversion of B73, displays consistent resistance to *S*. *hermonthica* across locations, seasons and diverse *Striga* ecotypes. This inbred line has therefore been used as a standard check in our breeding program to characterize maize inbred lines under artificial *Striga* infestation. Nonetheless, the use of temperate germplasm for resistance to *S. hermonthica* has been limited in tropical maize breeding programs. Assessment of the potential usefulness of elite temperate maize inbred lines can thus facilitate efforts toward their incorporation into elite maize inbred lines to enhance genetic gain in resistance against *S. hermonthica*.

Genetic improvement for adaptation and desirable agronomic traits is driven not only by access to adequate genetic variability but also by the quality of the genetic factors derived from donor parents [11]. The readily available expired industry inbred lines that had been commercially used for 20 years (Ex-PVP inbred lines) are potential sources of desirable alleles for high grain yield potential, earliness, desirable plant type, low ear placement, upright leaves, good standability, tolerance to abiotic stresses and resistance to diseases and insects [12–14]. These desirable traits can be introgressed into tropical elite maize inbred lines through backcrossing without reducing the frequency of existing favorable *S. hermonthica* resistance alleles. Studies in maize have demonstrated that exotic germplasm has been a reservoir of genes for broadening the genetic base of adapted inbred lines and increasing grain yield potential in tropical hybrids [6, 15–18]. Introgression of genes from exotic germplasm can also protect elite genotypes against new biotic and abiotic stresses and increase their nutrient-use-efficiency [19, 20]. Evaluating the usefulness of the Ex-PVP inbred lines as a potential source of novel beneficial alleles is thus critical for *S. hermonthica* resistance breeding to enhance genetic gain in maize at farm level.

The value of the Ex-PVP inbred lines as sources of desirable traits is difficult to directly predict because they have not been bred for adaptation to tropical production zones where specific foliar diseases and insects pressures are severe and climatic conditions are unpredictable. Understanding the combining ability of the temperate inbred lines with tropical *S. hermonthica* resistant inbred lines could then be useful to successfully incorporate the temperate inbred lines in a breeding program to enhance the genetic gains in hybrids targeted to the tropics where the parasite is endemic. The breeding value of the Ex-PVP inbred lines with diverse genetic backgrounds can thus be assessed in crosses with adapted inbred line testers representing the existing heterotic groups. Such a mating scheme generates information about the general and specific combining ability effects (GCA and SCA) of the inbred lines [21]. The resulting GCA and SCA effects of the Ex-PVP inbred lines can then be used to deploy complementary methods for classifying the inbred lines into heterotic groups [22, 23]. Furthermore, the genetic distance (GD) estimates from Diversity Array Technology (DArT) markers [24, 25] may also be useful to assign the Ex-PVP inbred lines into heterotic groups in a cost-effective manner [26]. Such an approach can provide complementary information to the yield-based assessment to select parental inbred lines for developing progenies with maximum variability in a breeding program [27].

IITA introduced many Ex-PVP maize inbred lines to improve agronomic performance of tropical *Striga* resistant and other elite inbred lines. Characterization of the heterotic affinities of the Ex-PVP temperate inbred lines to the existing elite tropical inbred lines is important for the identification and systematic introgression of temperate inbred lines into existing heterotic groups to develop parental inbred lines of hybrids with superior agronomic performance and resistance to *S*. *hermonthica* [28]. These studies were, therefore, conducted to (i) determine the usefulness of the Ex-PVP inbred lines for use as parents to improve grain yields and other desirable agronomic traits under artificial field infestation and non-infested conditions, (ii) define the heterotic affinities of the Ex-PVP inbred lines using two tropical *Striga* resistant testers, and (iii) assess the extent of genetic diversity among the Ex-PVP inbred lines and their divergence from the two testers using DArTag markers.

## Results

### Testcross performance

The combined analysis of variance revealed a significant environmental and hybrid effects on grain yield and other agronomic traits under *Striga*-infested and non-infested conditions and across multiple test locations (Table 1). The GCA mean squares for Ex-PVP inbred lines were significant for all the traits recorded under *Striga*-infested and non-infested conditions and across multiple test locations. Moreover, the GCA mean squares for the testers were significant for all or most traits recorded under both *Striga*-infested and non-infested conditions as well as across multiple test locations. The SCA effects (line x tester) were significant for grain yield, plant height and ear aspect under *Striga*-infestation, and for grain yield, days to anthesis, days to silking, and ear height under non-infested conditions. The SCA effects were significant for all measured traits across multiple test locations. The line and tester GCA mean squares were larger than those of the SCA mean squares for almost all the traits recorded under the three testing conditions. The line x environment and tester x environment interactions were not significant for grain yield and most agronomic traits under *Striga*-infested and non-infested conditions, whereas they were significant across multiple test locations. The tester x environment interaction was significant only for grain yield under *Striga* infestation. There was no significant line x tester x environment interaction for all measured traits under *Striga*-infested and non-infested conditions and across multiple test locations (Table 1). Repeatability values for measured traits varied from 0.55 to 0.81 under *Striga* infestation, from 0.49 to 0.90 under non-infested conditions and from 0.65 to 0.93 across multiple test locations (Table S1). Estimates of narrow-sense heritability varied from 0.20 to 0.73 under *Striga* infestation, from 0.32 to 0.73 under non-infested conditions and from 0.33 to 0.80 across multiple test locations (Table S1).

**Table 1:** Mean squares from the analysis of variance of grain yield and agronomic traits of testcrosses of 24 Ex-PVP maize inbred lines evaluated in 2020 and 2021 under *Striga*-infested, *Striga* non-infested, and across multiple test locations in Nigeria

Source of variation	DF	Grain yield	*Striga* damage^a^	Emerged *Striga* count^a^	Days to anthesis	Days to silking	Plant height	Ear height	Ear aspect
***Striga-infested***									
Environment (Env)	3	122,335,306.8†	98.5†	11,269.52†	863.85†	659.68†	18,515.6†	11,695.48†	0.57**
REP (Env)	4	2,694,269.2***	25.62†	206.12	26.8†	18.07†	976.45†	623.99†	0.14
Block (Env × Rep)	96	986,111.1†	1.4**	294.37*	3.39**	3.75	244.74***	154.38†	0.15*
Hybrid (H)	51	2,603,929.2†	2.78†	562.94†	6.33†	6.9†	313.82†	57.22	0.31†
Line (GCA)	23	2,318,317.1†	3.44**	668.53†	14.76†	16.34†	449.46***	123.5	0.31†
Tester (GCA)	1	76,415,103.6†	109.44†	1197.09*	14.64*	2.04	10,478.73†	112.73	0.17
Line × Tester (SCA)	23	1,510,019.5**	0.89	172.62	3.35	4.12	328.83*	55.87	0.21*
Hybrid × Env	153	711,692.3**	0.91	239.5	2.02	2.47	113.11	68.09	0.17**
Line × Env	69	779,993.7	1.16	134.78	2.58	2.74	159.58	135.37	0.3†
Tester × Env	3	3,582,002.6**	4.66*	489.93	7.29	3.02	544.25*	231.87	0.11
Line × Tester × Env	69	720,595.4	0.85	204.78	2.36	2.68	147.14	71.58	0.17
Error	108	451,673.1	0.8	200.58	2.04	2.7	13,128.66	48.73	0.1
Repeatability		0.81	0.79	0.66	0.81	0.79	0.73	0	0.55
CV (%)		24.41	14	65	2.5	2.8	6.99	10.15	10.8
Source of variation	DF	Grain yield	Days to anthesis	Days to silking	Plant height	Ear height	Ear aspect	Plant aspect	
***Striga non-infested***									
Environment (Env)	5	903,160,592†	1397.35†	1478.52†	145,453.01†	29,314.14†	3.4†	5.86†	
REP (Env)	6	16,528,463†	24.4†	25.65†	2058.44†	1193.58†	0.33*	1.55†	
Block (Env × Rep)	144	1,749,268**	2.81***	3.39***	335.66***	203.54†	0.14	0.2	
Hybrid (H)	51	4,406,667†	13.48†	14.26†	533.03†	239.63†	0.68†	0.41***	
Line (GCA)	23	9,373,757†	19.31†	18.56†	843.76†	283.43*	1.17†	0.61†	
Tester (GCA)	1	24,317,974***	159.39†	82.93†	14,410†	491.36	3.21†	3.35***	
Line × Tester (SCA)	23	2,890,278*	4.47*	5.63*	309.84	300.61**	0.18	0.34	
Hybrid × Env	255	1,208,517	2.19*	2.26	191.3	108.22	0.13	0.28**	
Line ×Env	115	1,888,242	3.12	3.49	244.28	172.34	0.14	0.29	
Tester × Env	5	3,397,689	10.41**	6.79	852.68*	176.23	0.65***	0.53	
Line × Tester × Env	115	1,315,022	2.24	1.99	199.52	132.81	0.16	0.25	
Error	162	1,065,614	267.08	1.9	196.6	95.17	0.12	0.17	
Repeatability		0.79	0.89	0.9	0.76	0.64	0.83	0.49	
CV (%)		19.35	2.29	2.41	7.34	12.21	12.69	15.26	
***Across multiple test locations***									
Environment (Env)	9	731,610,903†	1110.18†	1116.71†	116,659.67†	25,238.34†	3.2†		
REP (Env)	10	10,994,785†	25.36†	22.61†	1625.65†	1051.18†	0.27*		
Block (Env × Rep)	240	1,444,005†	3.04†	3.53***	299.3†	191.25†	0.15*		
Hybrid (H)	51	5,814,128†	18.6†	19.56†	681.73†	215.89†	0.78†		
Line (GCA)	23	8,745,093†	32.34†	32.61†	1145.12†	312.89**	1.18		
Tester (GCA)	1	87,217,218†	148.83†	†63.44	24,857.81†	195.02	1.5***		
Line × Tester (SCA)	23	3,474,128†	5.66**	7.15**	457.67*	290.19**	0.25**		
Hybrid × Env	459	1,062,000*	2.03	2.25	162.59	97.96	0.15**		
Line ×Env	207	1,642,063*	2.79	3.1	205.04	155.96	0.2		
Tester × Env	9	4,561,533***	11.01**	7.19*	654.78**	218.23	0.67		
Line × Tester × Env	207	1,071,283	2.27	2.29	180.12	114.89	0.16*		
Error		821,877	1.8	2.22	167.2	83.72	0.11		
Repeatability		0.86	0.93	0.93	0.85	0.64	0.83		
CV (%)		21.06	2.38	2.58	7.26	11.86	12.05		

*DF* Degree of freedom, ^a^10 weeks after planting

^*^, **, ***, † Significant at probability < 0.05, 0.01, 0.001 and 0.0001 levels, respectively

**Fig. 1:**
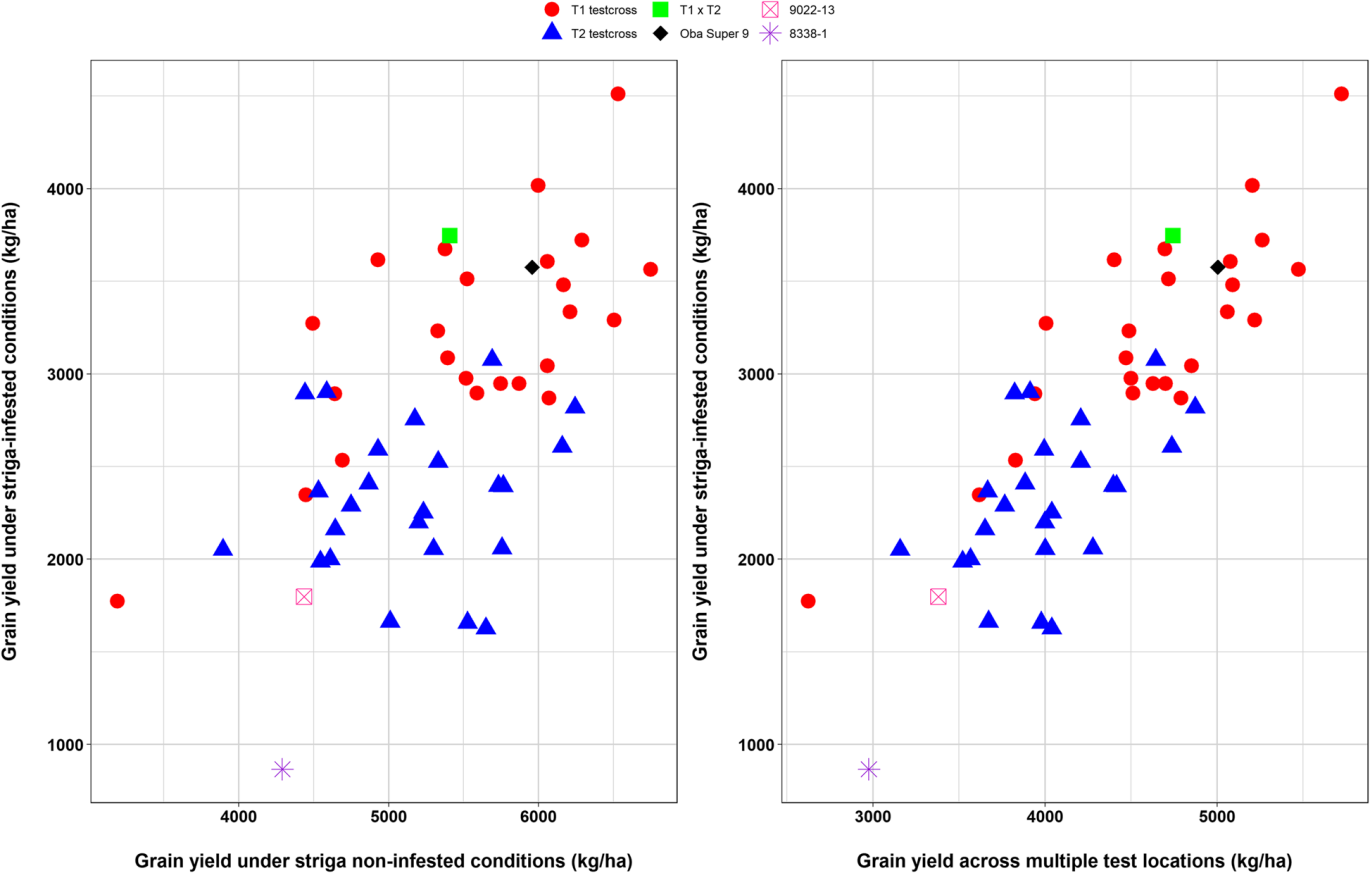
Distribution of grain yield under *Striga*-infested conditions, *Striga* non-infested conditions, and across multiple test locations for tester 1 testcrosses (T1 testcrosses), tester 2 testcrosses (T2 testcrosses), a cross between the testers (T1 x T2), a commercial *Striga *resistant check (Oba Super 9), a *Striga* tolerant check (9022-13), and a *Striga* susceptible check (8338-1)

The tolerant (9022–13) and susceptible (8338–1) hybrids were included as standard checks in the trial to determine the extent of damage caused by *S. hermonthica*. As shown in Table S1, 8338–1 sustained a grain yield loss of 80%, whereas 9022–13 sustained a grain yield loss of 59%. The cross between the two testers (T1 x T2) and the *Striga* resistant commercial hybrid (Oba Super 9) sustained grain yield losses of 31% and 40%, respectively. These results indicate that *Striga* infection was severe during the evaluation of the testcrosses. Twenty-one testcrosses of Ex-PVP inbred lines with T1 (HGA) and five testcrosses of Ex-PVP inbred lines with T2 (HGB) produced significantly higher grain yields than 9022–13 under *Striga* infestation (Fig. 1). None of the 48 testcrosses yielded significantly less than 9022–13 under *Striga* infestation. Also, 18 testcrosses of Ex-PVP inbred lines with T1 and a testcross between an Ex-PVP inbred line and T2 had mean grain yields that did not differ significantly from that of the T1 x T2 testcross. Five Ex-PVP inbred lines (HB8229-1, LH132-1, LH208-1, PHW79-1, and WIL900-1) generated high yielding testcrosses with both T1 and T2 under *Striga* infestation. All the T1 and T2 testcrosses that produced significantly higher grain yields than 9022–13 sustained similar or less *Strig*a damage and supported similar or less emerged *Striga* plants than 9022–13 (Table S1). Overall, the best testcrosses involving the same five Ex-PVP inbred lines (HB8229-1, LH132-1, LH208-1, PHW79-1, and WIL900-1) had similar or earlier anthesis and silking dates, similar or shorter plant and ear heights and desirable plant and ear aspect scores under infestation.

Under *Striga* non-infested conditions, 17 testcrosses of T1 and eight testcrosses of T2 produced significantly higher grain yield than 9022–13 (Table S1). Also, the 17 testcrosses of T1 and two testcrosses of T2 were amongst those that significantly out-yielded 9022–13 under *Striga* infestation. Only four testcrosses (PHP55-1 × T1, IBC2-1 × T1, PHP55-1 × T2 and IBC2-1 × T2) produced significantly lower grain yields than the T1 x T2 testcross. It is interesting to note that three testcrosses of T1 (PHW79-1 × T1, WIL900-1 × T1, and G80-1 × T1) produced significantly higher grain yields than T1 x T2 under non-infested conditions (Fig. 1, Table S1). In addition, two of these testcrosses (WIL900-1 × T1 and PHW79-1 × T1) produced significantly higher grain yields across multiple test locations. Amongst all testcrosses, only two (PHT177-1 × T1 and WIL900-1 × T1) showed positive standard heterosis of 7 to 21% for grain yield under *Striga* infested and non-infested conditions and across multiple test locations (Table S2). The positive standard heterosis of the testcross WIL900-1 × T1 was higher than that of the commercial hybrid check, Oba Super 9, which had negative standard heterosis for grain yield under *Striga* infested conditions (-5%) and positive standard heterosis under *Striga* non-infested conditions (10%) and across multiple test locations (6%). Although most of the testcrosses involving the two testers had positive standard heterosis for *Striga* damage rating and *Striga* count, WIL900-1 × T1 had 0 or negative standard heterosis for these traits. However, 17 testcrosses of T1 and seven testcrosses of T2 showed markedly less standard heterosis for *Striga* damage rating (varied from 3 to 35%) and *Striga* count (-43 to 95%) relative to 9022–13 (45% and 165%, respectively) and Oba Super 9 (10% and 239%, respectively).

### Combining ability of the Ex-PVP lines

GCA effect estimates of the Ex-PVP inbred lines for grain yield and two important traits under *Striga*-infestation and the GCA estimates for grain yield recorded under non-infested conditions and across multiple test locations are presented in Table 2. We found three Ex-PVP inbred lines, namely LH128-1, ICI 893–1 and PHKE6-1 showing positive but not significant GCA effects for grain yield under *Striga* infestation. Inbred lines PHR61-1, MDF-13D-1, HBA1-1 and LH214-1 combined small but positive GCA effect for grain yield under *Striga* infestation with large positive GCA effect under non-infested conditions and across multiple test locations. Overall, six inbred lines (PHR47 -1, LH208-1, PHW79-1, PHT177-1, HB8229-1 and WIL900-1) displayed moderate to significant positive GCA effects for grain yield under *Striga* infested and non-infested conditions and across multiple test locations (Table 2). Amongst these, inbred line WIL900-1 showed significant positive GCA effects for grain yield and negative GCA effects for *Striga* damage rating and *Striga* count. Likewise, inbred line HB8229-1 combined positive GCA effects for grain yield with negative GCA effect for *Striga* damage rating and *Striga* count. The two testers (T1 and T2) had contrasting GCA effects for grain yield under each testing condition. T1 displayed significant positive GCA effects for grain yield under *Striga*-infested (446 kg/ha, *p* < 0.05), *Striga* non-infested (213 kg/ha, *p* < 0.05) and across multiple test locations (305 kg/ha, *p* < 0.05), whereas T2 had significant negative GCA effects under *Striga*-infested (-446 kg/ha, *p* < 0.05), *Striga* non-infested (-212 kg/ha, *p* < 0.05) and across multiple test locations (-305, *p* < 0.05). In addition, 4 to 6 Ex-PVP inbred lines had significant and negative GCA effects for days to anthesis, days to silking and plant height under *Striga* infested and non-infested conditions and across multiple test locations (Table S3). Ex-PVP inbred lines G80-1, PHKE6-1, and PHT177-1 having positive GCA effects for grain yield under *Striga* infested or non-infested conditions showed negative GCA effects for days to anthesis and silking. Amongst all the testcrosses involving the Ex-PVP inbred lines, 15 had positive SCA effects for grain yield with T1 but negative SCA effects with T2 under *Striga* infestation (Table S4). Moreover, 11 Ex-PVP inbred lines had positive SCA effects for grain yield with T1 but negative SCA effects with T2 under non-infested conditions.

**Table 2:** General combining ability (GCA) effects of 24 Ex-PVP inbred lines for grain yield and two other traits under artificial *Striga* infestation and grain yield under non-infested conditions and across multiple test locations (MTL)

Line	Grain yield			*Striga* damage rating (10WAP)	*Striga* count (10WAP)
	Infested	Non- infested	Across MTL		
	(kg/ha)	(kg/ha)	(kg/ha)	(1–9)	(number)
PHKE6-1	245.58	-525.59	-216.04	0.04	-3.03
PHP55-1	-147.47	-758.18**	-533.13**	-0.08	-5.71*
PHR47 -1	218.38	399.54	328.15	0.23	15.4†
PHR61-1	8.91	653.4*	396.68*	-0.01	5.84*
PHT11 -1	-520.87*	498.61	91.9	0.73**	8.46**
PHT177-1	339.6	238.48	280.01	0.48	0.96
PHW53-1	-7.89	-322.19	-195.39	0.35	-1.65
PHW79-1	277	677.8*	518.56**	0.16	3.28
WIL900-1	1025.6†	749.18**	860.83†	-0.83**	-7.71**
WIL901-1	1.06	-22.35	-11.91	-0.33	-1.53
G80-1	-291.68	397.03	122.63	-0.14	-5.15
HB8229-1	422.65	14.99	179.14	-1.14†	-3.09
HBA1-1	88.78	554.76*	369.45	0.1	1.59
IBC2-1	-856.34***	-1818.07†	-1432.3†	0.41	-3.96
ICI 893–1	222.66	-630.61*	-288.22	-0.01	-5.96*
LH128-1	149.49	-349.79	-149	0.16	-5.4
LH132-1	-117.16	-606.46*	-409.66*	0.04	-4.4
LH208-1	233.55	534.07	414.94*	0.54*	7.09*
LH213-1	-418.15	431.79	92.89	0.73**	13.34†
LH214-1	96.35	626.86*	415.74*	-0.39	4.53
LH217-1	-474.62*	-365.46	-408.04*	-0.2	-2.15
LH51-1	-280.49	-297.2	-289.44	-0.39	-7.65**
MBST-1	-232.36	-391.13	-326.54	-0.64*	-6.21*
MDF-13D-1	17.43	278.93	175.41	0.16	3.15
GCA SE (L)	216.14	274.58	198.34	0.26	2.84
GCA SE (T)	96.58	76.8	68.93	0.11	1.12

^*^, **, ***, † significant at probability < 0.05, 0.01, 0.001 and 0.0001 levels, respectively

### Classifying Ex-PVP lines into heterotic groups using SCA effects and testcross yields

The mean grain yield and SCA effects of the Ex-PVP inbred lines in crosses with the two testers were used to classify the inbred lines into heterotic groups, namely HGA and HGB (Table S4). Under *Striga* infestation, 11 Ex-PVP inbred lines having grain yields that did not differ significantly from that of T1 x T2 testcross and displaying more than 100 kg/ha positive SCA effects with T1 were assigned to HGB (Table S4). Ex-PVP inbred lines PHT11 -1, LH214-1, PHW53-1 and LH51-1 in crosses with T1 that showed significantly lower grain yield than T1 x T2 testcross or displayed less than 100 kg/ha positive SCA estimates were not assigned to any heterotic group. The remining Ex-PVP lines with positive SCA effects in crosses with T2 but produced significantly less grain yields than the T1 x T2 testcross were not assigned to any heterotic group. Under non-infested conditions, 10 Ex-PVP inbred lines in crosses with T1 that produced as high as or significantly higher grain yields than the T1 x T2 testcross and showed more than 100 kg/ha positive SCA effects with T1 were classified into HGB (Table S4). Similarly, seven Ex-PVP inbred lines with positive SCA effects in crosses with T2 and with mean grain yields that did not differ significantly from that of the T1 x T2 testcross were assigned to HGA. The remaining seven Ex-PVP inbred lines showing less than 100 kg/ha positive SCA estimates in crosses with either T1 or T2 were not classified into any heterotic group. In multiple test locations, 10 Ex-PVP inbred lines that displayed positive SCA effects in crosses with T1 and did not differ significantly in mean grain yields compared to that of the T1 x T2 testcross were classified to HGB (Table S4). Similarly, three Ex-PVP inbred lines in crosses with T2 having positive SCA effects and producing grain yields not significantly different form that of the T1 x T2 testcross were classified to HGA. The remaining 11 inbred lines with less than 100 kg/ha positive SCA estimates either with T1 or T2 and producing grain yields that were significantly lower than that of the T1 x T2 testcross were not assigned to any group. Ex –PVP inbred lines G80-1, LH128-1, LH213-1, MBST-1, PHR47 -1, PHT177-1, PHW79-1 and WIL900-1 were consistently assigned to HGB under infested and non-infested conditions as well as across multiple test locations (Table S4). Also, three Ex-PVP inbred lines (HBA1-1, PHR61-1 and PHKE6-1) were consistently assigned to HGA under non-infested conditions and across multiple test locations.

The HSGCA effects for grain yield of the Ex-PVP inbred lines in combination with the two testers were also used as a complementary method to classify the inbred lines into heterotic groups, namely HGA and HGB (Table 3, Table S4). Using the criteria described in the materials and methods, 15 Ex-PVP inbred lines were assigned to HGB, whereas 9 Ex-PVP inbred lines were assigned to HGA under *Striga* infestation (Table 3, Table S4). Under non-infested conditions, HGB consisted of 13 Ex-PVP inbred lines, whereas HGA contained 8 inbred lines with the remaining three inbred lines not assigned to any group. Across multiple test locations, 15 Ex-PVP inbred lines were placed into HGB and eight Ex-PVP inbred lines into HGA with one inbred line not assigned to any group. It is interesting to note that 10 Ex-PVP inbred lines were assigned to HGB and six inbred lines were assigned to HGA under *Striga*-infested and non-infested conditions as well as across multiple test locations (Table 3, Table S4). Assigning the Ex-PVP inbred lines into heterotic groups using HSGCA appears to be better than grouping the inbred lines based on SCA and grain yield (Table S4).

**Table 3:** Heterotic grouping of 24 Ex-PVP inbred lines based on heterotic group’s specific and general combining ability (HSGCA) for grain yield under *Striga* -infested, *Striga* non-infested, and across multiple test locations

HGA (T1)	HGB (T2)	Not classified
*Striga-infested*		
WIL901-1, HB8229-1, PHP55-1, LH132-1, LH217-1, PHR61-1, PHKE6-1, HBA1-1, IBC2-1	PHT11 -1, LH214-1, ICI 893–1, PHW53-1, LH213-1, LH51-1, G80-1, PHW79-1, PHR47 -1, PHT177-1, WIL900-1, LH128-1, MBST-1, LH208-1, MDF-13D-1	-
*Striga non-infested*		
LH208-1, MDF-13D-1, LH132-1, LH217-1, PHR61-1, PHKE6-1, HBA1-1, IBC2-1	PHW53-1, LH213-1, LH51-1, G80-1, PHW79-1, PHR47 -1, PHT177-1, WIL900-1, LH128-1, MBST-1, WIL901-1, HB8229-1, PHP55-1	LH214-1, PHT11 -1, ICI 893–1
*Across multiple test locations*		
PHP55-1, MDF-13D-1, LH132-1, LH217-1, PHR61-1, PHKE6-1, HBA1-1, IBC2-1	PHT11 -1, LH214-1, ICI 893–1, PHW53-1, LH213-1, LH51-1, G80-1, PHW79-1, PHR47 -1, PHT177-1, WIL900-1, LH128-1, MBST-1, HB8229-1, LH208-1	WIL901-1

### Assigning Ex-PVP inbred lines into heterotic groups using DArTag markers

The 24 Ex-PVP inbred lines and the two testers were genotyped with 3,305 DArTag SNP markers, and a total of 2,053 were finally retained after quality assessment. The markers were distributed across the entire maize genome, with the highest number of markers located on chromosome 5 (Fig. S1). The average gene diversity was 0.40 and varied from 0.39 to 0.41. The PIC values were uniformly distributed across the 10 chromosomes, with an average of 0.31. Heterozygosity varied from 0.03 to 0.05 with an average of 0.04. The mean major allele frequency was 0.69 with similar values across the 10 chromosomes (Fig. S1).

The *Striga* resistant testers representing two heterotic groups in the IITA’s maize breeding program had a genetic distance of 0.69 (Table S5). The genetic distances estimate between pairs of the Ex-PVP inbred lines with tester T1 varied from 0.62 to 0.83 with an average of 0.80, whereas those with T2 varied from 0.69 to 0.83 with an average of 0.79 (Table S5). The highest genetic distance estimates were found between T1 and three Ex-PVP inbred lines (PHP55-1, LH217-1, and LH51-1) and between T2 and three Ex-PVP inbred lines (LH213-1, LH214-1, and LH51-1), while the lowest genetic distance estimate was between MDF-13D-1 and T1 (Table S5).

**Fig. 2:**
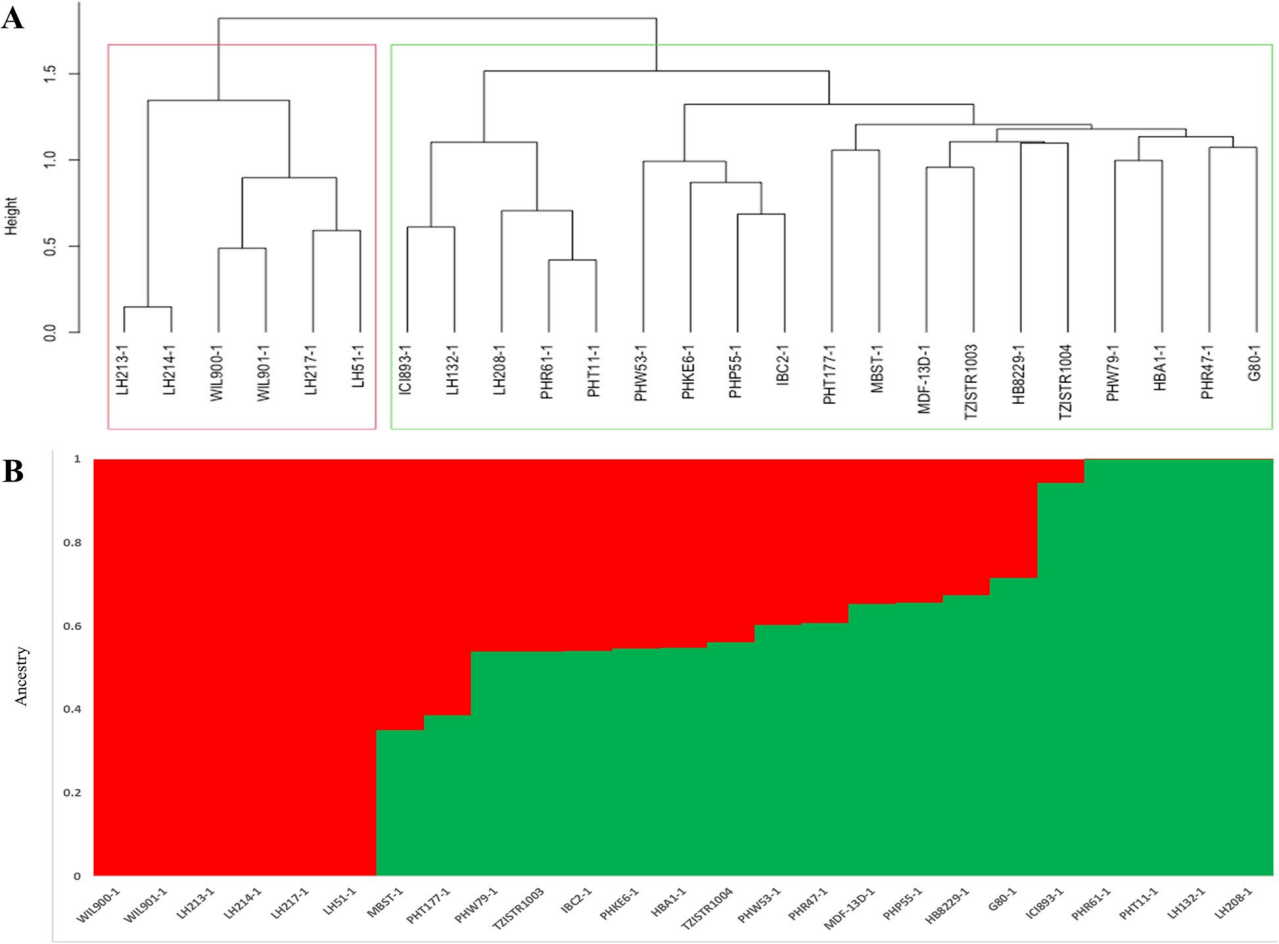
(A) Clustering of 23 Ex-PVP inbred lines and two testers using Ward's method. (B) Estimated population structure of the Ex-PVP inbred lines as revealed by the 2053 SNP markers for K = 2. Cluster 1 and cluster 2 are coloured with red and green, respectively

**Fig. 3:**
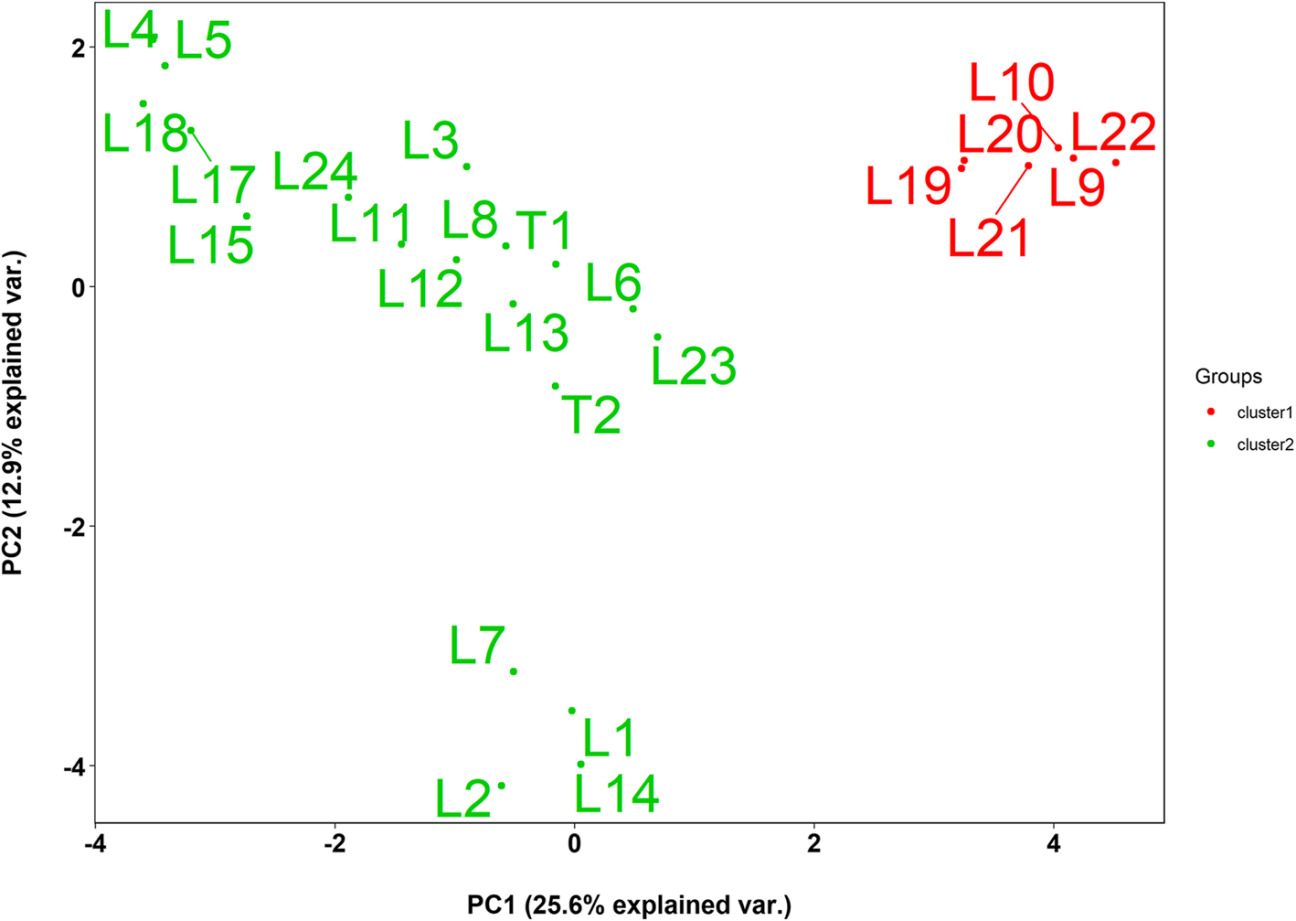
Principal Component Analysis (PCA) for 23 Ex-PVP inbred lines and the two testers

The Ward’s minimum variance hierarchical cluster method separated the Ex-PVP inbred lines into two main clusters, with the second cluster further divided into two sub-clusters (Fig. 2A). The first cluster contained six Ex-PVP inbred lines, whereas the second cluster was composed of the remaining Ex-PVP inbred lines and the two testers. This is surprising because the two testers do not share a common parentage with the Ex-PVP inbred lines. It is interesting to note that five of the six Ex-PVP inbred lines (WIL900-1, LH51-1, LH213-1, LH214-1 and WIL901-1) that were assigned to the first cluster had positive SCA effects under *Striga* infested and non-infested conditions and across multiple test locations in combination with T1 (Table S4). The genetic distances of these inbred lines with the two testers were also very high (Table S5). Furthermore, some high yielding Ex-PVP inbred lines in crosses with T1 were grouped together with the testers (Fig. 2A). In structure analysis, the Ex-PVP inbred lines were divided into two clusters considering the rapid elbow at K = 2 (Fig. S2, Fig. 2B). The number of major clusters and the corresponding number of Ex-PVP inbred lines grouped together using structure analysis was similar to the Ward’s hierarchical cluster analysis. Likewise, principal component analysis revealed two major groups with the first two principal component axes (PC1 and PC2) accounting for 26% and 13% of the total molecular variation among inbred lines, respectively (Fig. 3). Similar to the hierarchical cluster analysis, the first group had six Ex-PVP inbred lines, while the second group has the remaining 17 Ex-PVP inbred lines and the two testers, with the second group further split into two sub-clusters. Therefore, the three clustering methods generated the same groups. However, structure analysis included 11 Ex-PVP inbred lines in the second sub-cluster (Table S3), with the remaining four Ex-PVP inbred lines and the two testers having membership probabilities below 60% assigned to the admixed group. It appears that yield-based assignment of the Ex-PVP lines to heterotic groups under infested and non-infested conditions and across multiple test locations were different from separating the Ex-PVP lines into groups using DArTag markers.

## Discussion

*Striga* resistant tropical maize germplasm is endowed with considerable genetic diversity for different agronomic traits recorded under both *Striga* infested and non-infested conditions [29]. Continual introgression of exotic germplasm as donors of desirable agronomic and adaptive traits absent in tropical germplasm can broaden and diversify the genetic base for increasing the rate of genetic gain in resistance breeding against the parasite. The Ex-PVP inbred lines that had been used as parents of commercialized hybrid represent promising sources of new beneficial alleles for recovering in genetic backgrounds adapted to *Striga* endemic tropical lowlands [14, 30]. Dubreuil et al. [31] emphasized the need for accurate assignment of inbred lines to heterotic groups for their efficient utilization in a breeding program. The current study was therefore conducted to determine the heterotic patterns of 24 Ex-PVP inbred lines using two tropical *Striga* resistant inbred testers and molecular markers. The results of our study clearly showed that *Striga* infection was severe during evaluation of testcrosses of the Ex-PVP inbred lines across locations and years. The line × tester × environment interactions were not significant for all measured traits under *Striga* infested and non-infested conditions as well as across multiple test locations notwithstanding the presence of significant environmental effects. These results indicated that the performance of the Ex-PVP inbred lines in crosses with testers was consistent across environments under the three diverse testing conditions, allowing the identification of promising Ex-PVP inbred lines for use in breeding and the assignment of the inbred lines to existing heterotic groups. The high repeatability estimates observed for each trait indicated a level of accuracy of field trials and a high proportion of genetic effects in the total variation observed for the traits. The high estimates of narrow-sense heritability recorded for the evaluated traits is an indication of the preponderance of additive gene effects in the inheritance of the traits.

Although the testcrosses evaluated in our study contained 50% temperate germplasm in their genetic backgrounds that usually affect performance in tropical lowlands, most of the testcrosses involving the T1 tester produced significantly higher grain yields than a standard tolerant single-cross hybrid check (9022–13) under *Striga* infestation and non-infested conditions and across multiple test locations. Moreover, many testcrosses between Ex-PVP inbred lines and T1 produced competitive grain yields compared to the cross between testers under *Striga* infestation. Ex-PVP inbred lines HB8229-1, LH132-1, LH208-1, PHW79-1, and WIL900-1 were parents of the highest yielding testcrosses with T1 and T2 under *Striga* infestation. Again, many testcrosses of the Ex-PVP inbred lines with T1 and some testcrosses with T2 produced either competitive or significantly better grain yields than the cross between testers under non infested conditions and across multiple test locations. These results indicate the potential of these Ex-PVP inbred lines to increase genetic diversity and grain yield following intensive selection through successive inbreeding generations. Furthermore, most of the testcrosses with positive heterosis for grain yield and negative heterosis for *Striga* damage and emerged *Striga* count involved T1 as a tester, indicating that the Ex-PVP inbred lines interacted positively with the genetic background of this *Striga* resistant tester. These results suggest that the Ex-PVP inbred lines possess beneficial alleles for increasing grain yields and other *Striga* resistance traits that can be transferred to *Striga* resistant IITA inbred lines [12–14, 18].

The present study showed that both additive and dominance gene action were important in regulating grain yield and other desirable agronomic traits recorded under *Striga* infested and non-infested conditions and across multiple test locations, with a predominance of additive genetic variance in this set of testcrosses. Our study identified Ex-PVP inbred lines HBA1-1, HB8229-1, LH214-1, LH208-1, MDF-13D-1, PHR47 -1, PHR61-1, PHT177-1, PHW79-1, and WIL900-1 displaying moderate to significant positive GCA effects for grain yield under *Striga* infested and non-infested conditions and across multiple test locations. Negative GCA effects are preferable for emerged *Striga* count and *Striga* damage rating because they are indicators of the capacity of parents to transmit higher levels of *Striga* resistance to their progeny. Although the EX-PVP inbred lines have not been exposed to *S. hermonthica* during their development, inbred lines WIL900-1 and HB8229-1 that combined positive GCA effects for grain yield with negative GCA effect for *Striga* damage rating and *Striga* count could be useful for improving *Striga* resistance in tropical maize hybrids. WIL900-1 was the best Ex-PVP inbred line in the present study because it had high grain yields in combination with T1 and combined significant positive GCA effects for grain yield with significant negative GCA effects for emerged *Striga* count and *Striga* damage rating under *Striga* infestation. Moreover, Ex-PVP inbred lines G80-1, PHKE6-1, and PHT177-1 that displayed negative GCA effects for days to anthesis and silking can transfer earliness to progeny when they are crossed to other parental inbred lines. Similarly, Bari and Carena [32] identified Ex-PVP inbred lines with beneficial alleles for improving agronomic performance and stress resilience in maize inbred lines. The results also suggest that the tester T1 has the capacity to reveal differences in performance among testcrosses of the Ex-PVP inbred lines, making it an ideal tester to characterize the breeding values of temperate inbred lines in maize breeding programs [33].

Breeding for *Striga* resistant hybrids depends on the identification and utilization of heterotic groups and heterotic patterns of inbred lines [28]. Assigning the Ex-PVP inbred lines to existing heterotic groups can thus facilitate their systematic introgression into *Striga* resistant tropical inbred lines to optimize heterosis in hybrids. In the present study, the yield-based SCA method coupled with HSGCA method were used to assign the Ex-PVP inbred lines to existing heterotic groups. The yield-based SCA method consistently assigned eight Ex-PVP inbred lines to HGA and three lines to HGB under *Striga* infestation and non-infested conditions and across multiple test locations. Similarly, the HSGCA method classified 11 Ex-PVP inbred lines to HGA and 7 inbred lines to HGB under the three growing conditions, indicating the effectiveness of the two grouping methods. Although the DArTag SNP markers revealed substantial divergence of the Ex-PVP inbred lines from the two testers, it did not assign the inbred lines into groups defined based on yield-based SCA and HSGCA, possibly due to the environmental effects on the yield performance of the testcrosses. Also, the traits recorded under the various growing conditions may not be linked to the DArTag SNP markers used for the cluster analysis. This is consistent with the results of Menkir et al. [23] that reported no match between yield-based-SCA defined heterotic groups and groups established using molecular markers. Moreover, Barata and Carena [34] found significant inconsistencies between molecular marker-based separation and field trial based separation of a diverse set of inbred lines. As suggested by Melchinger [35], the heterotic groups defined based on testcross performance in our study could facilitate the successful introgression of the Ex-PVP inbred lines into the existing *Striga* resistant heterotic groups in IITA.

## Conclusions

This study identified U.S. Ex-PVP maize inbred lines that could be used to improve the agronomic performance of *Striga* resistant tropical germplasm. The inbred lines with outstanding grain yield in crosses with the testers and good positive GCA effects for grain yield could be used to enhance the grain yield performance of tropical germplasm. The identified inbred lines with significant negative GCA effects for emerged *Striga* count and *Striga* damage rating could serve as source of favourable alleles for improving *Striga* resistance in tropical maize germplasm. The information on the heterotic affinities of the Ex-PVP inbred lines and their genetic distances in relation to the tropical maize inbred testers can guide the selection of parental inbred lines for generating backcrosses to develop parental inbred lines with optimum expression of heterosis in hybrids.

## Materials and Methods

### Plant material and Experimental design

Twenty-four Ex-PVP maize inbred lines (Table 4), which were selected based on erect leaves, small tassels, upright leaves, resistance to lodging, low ear placement and uniform arrangements in kernel rows during seed increases at Ibadan in 2018, and two testers were used in this study. The Ex-PVP numbers in Table 4 can be used to find more information about these inbred lines at the Agricultural Research Services of Germplasm Resources Information Network (GRIN) web page of USDA (https://www.ars-grin.gov/Pages/Collections, accessed on 09 February 2020). The two testers, TZISTR1003 (T1) and TZISTR1004 (T2), were tropical *Striga* resistant inbred lines belonging to two heterotic groups developed at IITA. T1 was derived from a broad-based tropical composite (TZL COMP1-W), whereas T2 was developed from a backcross containing *Zea diploperennis* (Zea-Diplo BC4) as donor of *Striga* resistance alleles. The formation of the two source populations has been described in detail by Kling et al. (1999). Using a line x tester mating design, 48 testcrosses were generated by crossing the 24 Ex-PVP inbred lines with each of the two testers in IITA’s research field at Ibadan (7°29′11.99″N, 3°54′2.88″E, altitude 190 masl) in Nigeria during the dry seasons (December 2019 to April 2020 and December 2020 to April 2021). The 48 testcrosses along with a cross between the two testers (T1 × T2), a tolerant (9022–13), a susceptible (8338–1) and commercial *Striga* resistant (Oba Super 9) hybrids used as checks were evaluated under artificial *Striga*-infested and *Striga* non-infested conditions as well as in three test locations in northern and southern guinea savanna of Nigeria viz; Saminaka (8°39´ E, 10°34´ N, 760 masl), Abuja (7°20´ E, 9°15´ N, 431 masl), and Mokwa (5°4´ E, 9°18´ N, 457 masl) for two seasons (June to November 2020 and June to November 2021).

**Table 4:** List of Ex-PVP inbred lines along with their PVP numbers and approximate heterotic groups and inbred testers used in the present study

Line	Pedigree	PVP Number	Heterotic group*	Origin
L1	PHKE6-1	9,300,111	Not defined**	Pioneer Hi-Bred International
L2	PHP55-1	8,900,318	Stiff-Stalk	Pioneer Hi-Bred International
L3	PHR47-1	8,800,213	Stiff-Stalk	Pioneer Hi-Bred International
L4	PHR61-1	9,100,100	Not defined	Pioneer Hi-Bred International
L5	PHT11-1	9,100,101	Amargo/Stiff-Stalk	Pioneer Hi-Bred International
L6	PHT77-1	8,800,038	Non-Stiff-Stalk	Pioneer Hi-Bred International
L7	PHW53-1	9,300,116	Not defined	Pioneer Hi-Bred International
L8	PHW79-1	8,800,220	Non-Stiff-Stalk	Pioneer Hi-Bred International
L9	WIL900-1	8,900,092	Not defined	Monsanto Technology
L10	WIL901-1	8,900,093	Non-Stiff-Stalk	Monsanto Technology
L11	G80-1	8,400,128	Stiff-Stalk	Pioneer Hi-Bred International
L12	HB8229-1	8,800,190	Stiff-Stalk	Dekalb Plant Genetics
L13	HBA1-1	8,500,069	Not defined	DeKalb-Pfizer Genetics
L14	IBC2-1	8,700,198	Non-Stiff-Stalk	DeKalb-Pfizer Genetics
L15	ICI 893–1	9,200,040	Not defined	Advanta Technology Limited
L16	LH128-1	9,100,067	Non-Stiff-Stalk	Holden's Foundation Seeds
L17	LH132-1	8,300,148	Stiff-Stalk	Holden's Foundation Seeds
L18	LH208-1	9,100,069	Stiff-Stalk	Holden's Foundation Seeds
L19	LH213-1	9,100,071	Non-Stiff-Stalk	Holden's Foundation Seeds
L20	LH214-1	9,100,266	Not defined	Holden's Foundation Seeds
L21	LH217-1	9,300,036	Not defined	Holden's Foundation Seeds
L22	LH51-1	8,200,062	Non-Stiff-Stalk	Holden's Foundation Seeds
L23	MBST-1	8,800,194	Non-Stiff-Stalk	DeKalb-Pfizer Genetics
L24	MDF-13D-1	8,200,151	Non-Stiff-Stalk	DeKalb-Pfizer Genetics
T1	TZISTR1003	-	Heterotic group A	IITA
T2	TZISTR1004	-	Heterotic group B	IITA

^*^ Heterotic groups were adopted from Mikel & Duley (2006) and GRIN website; ** Means not clearly indicated or identified in the PVP documents

The trial was arranged in a 13 × 4 alpha-lattice design with two replications. Experimental plots were single rows each 4 m long, with a plant-to-plant spacing of 0.25 m within a row, and row spacing of 0.75 m. The population density was 53,000 plants ha^−1^ keeping only one plant per hill. The arrangement of infested and non-infested strips for each hybrid were done following the method described by Menkir et al. [36]. In each location, *S. hermonthica* seeds were collected from sorghum fields in the previous season for infestations. Suicidal germination of existing *Striga* seeds was elicited by injecting ethylene gas into the soil of each non-infested plot. NPK 15–15-15 fertilizer was applied at the rate of 60 kg ha^−1^. Weeds other than *S. hermonthica* were manually removed from plots.

### Agronomic Data Collection

Data were collected from both infested and non-infested plots on plant height (PHT), days to anthesis (DYANTH), days to silking (DYSK), ear aspect (EASP), grain weight and grain moisture. Data on Ear height (EHT), plant aspect (PASP), and husk cover (HUSK) were only collected from non-infested plots. PHT and EHT were measured in cm as the distance from the base of the plant to the first tassel branch and the node bearing the upper ear, respectively. DYANTH and DYSK were recorded as the number of days from planting to the date when 50% of the plants in a plot have tassels shedding pollen and had emerged silks, respectively. Anthesis-silking interval (ASI) was calculated as the difference between DYSK and DYANTH. HUSK was scored based on a 1 to 5 scale, where 1 represented husk tightly arranged and extend beyond the ear tip and 5 represented loose and exposed husk tip. Ear aspect was scored on 1 to 5 scale, where 1 represented clean, well filled, uniform and larger ears, while 5 represented diseased, poorly filled, variable and smaller ears. Plant aspect was also scored on a 1 to 5 scale, where 1 represented uniform, clean, vigorous and good overall phenotypic appeal, while 5 represent weak, diseased and poor overall phenotypic appeal. Harvested ears were shelled and grain moisture content of shelled grains was measured using a portable Dickey-John moisture tester. The grain weight and moisture content were used to compute grain yield adjusted to 15% moisture. In addition, host plant damage symptoms rating and the number of emerged *S. hermonthica* plants were recorded at 8 and 10 weeks after planting (WAP) in the *Striga* infested plots at Mokwa and Abuja. *Striga* damage was scored per plot on a 1 to 9 scale where 1 = no visible host plant damage symptom, and 9 = all leaves completely scorched, resulting in premature death [4].

### DArTag genotyping

Leaves samples were collected from 10 seedlings of each inbred lines and the testers three weeks after planting. The leaves were freeze-dried using Labconco Freezone 2.5L system lyophilizer (Marshall Scientific, USA) after which leaf discs sampled into 96-well strips were sent to the Diversity Arrays facility, Canberra, Australia (https://www.diversityarrays.com/ accessed on 24 November 2021) for DNA extraction and targeted genotyping with a proprietary maize SNP DArTag assay (https://www.diversityarrays.com/technology-and-resources/targeted-genotyping/ accessed on 25 March 2022). DArTag is a genotyping technology that amplifies selected SNPs discovered by DArTseq [37] and genotyping by sequencing methods. The SNPs were captured using a single oligonucleotide and the target region containing the sequence variants were amplified while attached to a sample specific barcode. The resulting libraries were sequenced and processed using DArT’s proprietary pipeline to produce the marker panel (https://www.diversityarrays.com/technology-and-resources/targeted-genotyping/ accessed on 25 March 2022).

### Data Analysis

A total of 3,305 DArTag markers were used for the genotyping. PowerMarker version 3.25 [38] was used to filter out markers with > 10% missing data, major allele frequency (MAF) > 95%, and heterozygosity > 20%. Finally, 2,053 markers were retained for further analyses. Summary statistics including MAF, polymorphic information content (PIC), gene diversity, and heterozygosity were computed with PowerMarker version 3.25 [38].

To understand the level of admixture within and among the inbred lines, population structure analysis was conducted through Admixture [39]. The analysis was carried out using the Bayesian Information Criterion (BIC). Cross-validation error (k) means ranging from k = 2 to k = 10 were analyzed to determine the optimal number of clusters. Inbred lines with membership probabilities equal or greater than 60% were assigned to the corresponding sub-group, while inbred lines with less than 60% membership probability were considered as admixed [40].

Jaccard’s dissimilarity matrix was generated using the entire SNP markers using the Jaccard method implemented in the phylentropy R package [41]. The distance matrix was then used to build Ward’s minimum variance hierarchical clusters using the Analyses of Phylogenetics and Evolution (ape) package [42] implemented in R [43]. Principal Component Analysis (PCA) was also computed in FactorMiner R package [44] to visualize the pattern of genetic dissimilarities within and between sub-groups. Silhouette method was used to estimate the maximum cluster for the PCA.

For the hybrid trials, each location-year combination was considered an environment while *Striga*-infested and *Striga* non-infested were considered as research conditions. Following the procedure for line $\times$ tester [45], combined analysis of variance (ANOVA) was performed for each and across multiple test locations using proc mixed procedure in SAS version 9.4 [46]. Hybrids were considered fixed effects, while environment, replication (environment), block (replication $\times$ environment), environment $\times$ hybrid were considered random effects in the linear model. Proc mixed fits a wide class of mixed models and incorporates random effects in the model.

Standard heterosis (H) was also calculated for each testcross using the formula of Fan et al. [47]:

$$H=100\% \times ({F}_{1}-CK)/CK$$

where F_1_ is the grain yield of a testcross and CK is the grain yield of the hybrid between the two testers (T1 × T2).

After exclusion of the checks, the GCA and SCA effects of the parental inbred lines and the variance components for each trait across multiple test locations were calculated with Analysis of Genetic Design (AGD-R, V.5.0) [48]. Restricted Maximum Likelihood Method (REML) was used to estimate the variance components [48].

For the SCA and yield-based heterotic grouping, we used the combining ability effects and mean grain yields of the Ex-PVP inbred lines in crosses with the two testers [23]. An inbred line having positive SCA effects with one of the testers and negative SCA effects with the other tester coupled with testcross mean grain yield not significantly different or greater than the mean grain yield of the T1 $\times$ T2 testcross was assigned to a specific heterotic group [23]. These testcross grain yield levels were considered appropriate considering the fact that the testcrosses have only 50% of their genetic backgrounds carrying favourable alleles controlling adaptive traits.

We also used the HSGCA method of Fan et al. [22] as a complementary method to classify the Ex-PVP inbred lines into heterotic groups. Using this method, any inbred line with positive HSGCA effects with T1 was assigned to HGB. Inbred lines with positive HSGCA effects with T2 were assigned to HGA. When an inbred line has either negative or positive HSGCA with both testers, we kept the inbred line with the heterotic group where it has the smallest positive or the largest negative HSGCA value.

## Supplementary Information

**Additional file 1: Table S1.** Mean performance of 24 Ex-PVP inbred lines testcrosses and checks for grain yield and other agronomic traits evaluated under Striga -infested, Striga non-infested conditions and across multiple test locations

**Additional file 2: Table S2.** Standard heterosis for grain yield, Striga damage, and emerged Striga count for 24 Ex-PVP inbred lines testcrosses and checks

**Additional file 3: Table S3.** General combining ability (GCA) effects of 24 Ex-PVP inbred lines for grain yield and other agronomic traits under artificial striga infestation, striga non-infested, and across multiple test locations

**Additional file 4: Table S4.** Mean grain yield, specific combining ability (SCA) effects, yield and SCA-based heterotic grouping and DArTag SNP-based groups of 24 Ex-PVP maize inbred lines under Striga -infested, Striga non-infested conditions and across multiple test locations

**Additional file 5: Table S5.** Genetic distances (identity-by-state, IBS) between the Ex-PVP maize inbred lines and the tropical Striga resistant inbred testers

**Additional file 6: Fig. S1.** Summary statistics of 2053 DArTag SNP markers used to assess the genetic diversity among the Ex-PVP inbred lines. **Fig. S2.** Determination of the most appropriate number of clusters in structure analysis using cross-validation error (k) means

## Data Availability

The DArTag datasets used in the present study have been deposited at the IITA repository. https://doi.org/10.25502/81by-4t56/d. Link to CKAN: https://data.iita.org/dataset/zea-ex-pvp-inbred-lines-and-tropical-inbred-testers-for-diversity-assessment-and-heterotic-grouping
