# Supplementary material for: Optimizing use of U.S. Ex-PVP inbred lines for enhancing agronomic performance of tropical Striga resistant maize inbred lines
Source: BMC Plant Biol. 2022 Jun 10;22:286. doi: 10.1186/s12870-022-03662-1 (PMC9185936; doi:10.1186/s12870-022-03662-1)
Supplement: Supplementary file 5 — Additional file 5: Table S5. Genetic distances (identity-by-state, IBS) between the Ex-PVP maize inbred lines and the tropical Striga resistant inbred testers [file 12870_2022_3662_MOESM5_ESM.docx]

**Table S5.** Genetic distances (Jaccard) between the Ex-PVP maize inbred lines and the tropical *Striga* resistant inbred testers

| Inbred lines | Testers | |
| --- | --- | --- |
|  | TZISTR1003 (T1) | TZISTR1004 (T2) |
| PHKE6-1 | 0.81 | 0.77 |
| PHP55-1 | 0.83 | 0.78 |
| PHR47 -1 | 0.79 | 0.78 |
| PHR61-1 | 0.81 | 0.81 |
| PHT11 -1 | 0.80 | 0.79 |
| PHT177-1 | 0.82 | 0.78 |
| PHW53-1 | 0.80 | 0.76 |
| PHW79-1 | 0.81 | 0.78 |
| WIL900-1 | 0.81 | 0.82 |
| WIL901-1 | 0.82 | 0.82 |
| G80-1 | 0.81 | 0.78 |
| HB8229-1 | 0.76 | 0.76 |
| HBA1-1 | 0.82 | 0.76 |
| IBC2-1 | 0.82 | 0.75 |
| ICI 893-1 | 0.81 | 0.80 |
| LH132-1 | 0.81 | 0.81 |
| LH208-1 | 0.81 | 0.80 |
| LH213-1 | 0.82 | 0.83 |
| LH214-1 | 0.82 | 0.83 |
| LH217-1 | 0.83 | 0.82 |
| LH51-1 | 0.83 | 0.83 |
| MBST-1 | 0.77 | 0.77 |
| MDF-13D-1 | 0.62 | 0.74 |
| TZISTR1003 |  | 0.69 |
| TZISTR1004 | 0.69 |  |
| **Min** | 0.62 | 0.69 |
| **Max** | 0.83 | 0.83 |
| **Mean** | 0.80 | 0.79 |
