# Supplementary material for: Optimizing use of U.S. Ex-PVP inbred lines for enhancing agronomic performance of tropical Striga resistant maize inbred lines
Source: BMC Plant Biol. 2022 Jun 10;22:286. doi: 10.1186/s12870-022-03662-1 (PMC9185936; doi:10.1186/s12870-022-03662-1)
Supplement: Supplementary file 6 — Additional file 6: Fig. S1. Summary statistics of 2053 DArTag SNP markers used to assess the genetic diversity among the Ex-PVP inbred lines. Fig. S2. Determination of the most appropriate number of clusters in structure analysis using cross-validation error (k) means [file 12870_2022_3662_MOESM6_ESM.pdf]

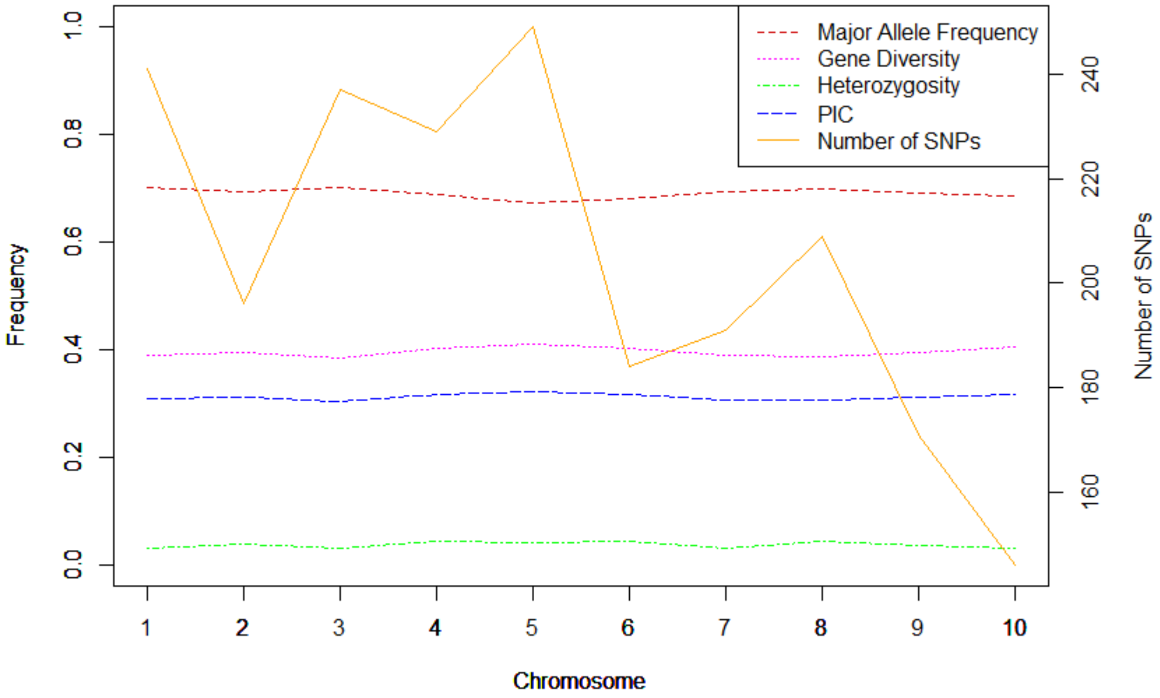

Fig. S1. Summary statistics of 2053 DArTag SNP markers used to assess the genetic diversity among the Ex-PVP inbred lines

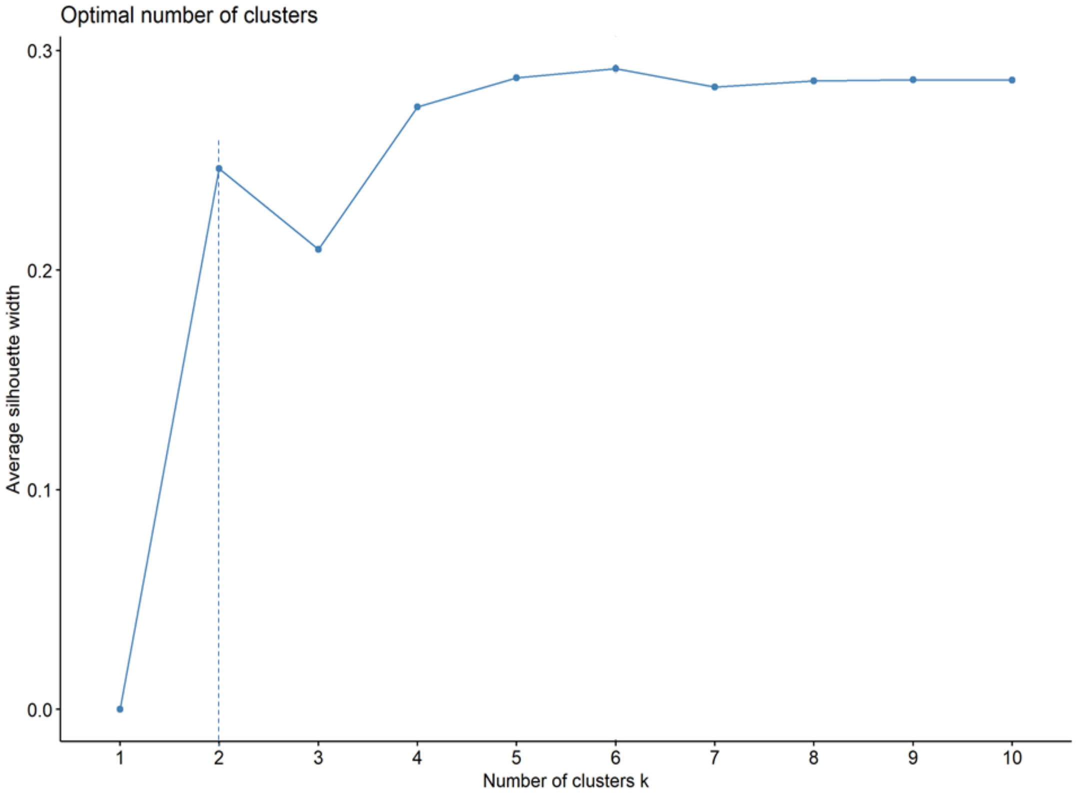

Fig. S2. Determination of the most appropriate number of clusters in structure analysis using cross-validation error (k) means
